# Uracil excision by endogenous SMUG1 glycosylase promotes efficient Ig class switching and impacts on A:T substitutions during somatic mutation

**DOI:** 10.1002/eji.201444482

**Published:** 2014-05-27

**Authors:** Felix A. Dingler, Kristin Kemmerich, Michael S. Neuberger, Cristina Rada

**Affiliations:** ^1^ MRC Laboratory of Molecular BiologyCambridgeUK; ^2^ Kristin Kemmerich, Human Health TherapeuticsNational Research Council Canada (NRC)100 Sussex DriveK1A 0R6OttawaONCanada

**Keywords:** Class switching, DNA deamination, Somatic hypermutation, Uracil

## Abstract

Excision of uracil introduced into the immunoglobulin loci by AID is central to antibody diversification. While predominantly carried out by the UNG uracil‐DNA glycosylase as reflected by deficiency in immunoglobulin class switching in *Ung^−/−^* mice, the deficiency is incomplete, as evidenced by the emergence of switched IgG in the serum of *Ung^−/−^* mice. Lack of switching in mice deficient in both UNG and MSH2 suggested that mismatch repair initiated a backup pathway. We now show that most of the residual class switching in *Ung^−/−^* mice depends upon the endogenous SMUG1 uracil‐DNA glycosylase, with in vitro switching to IgG1 as well as serum IgG3, IgG2b, and IgA greatly diminished in *Ung^−/−^Smug1^−/−^* mice, and that *Smug1* partially compensates for *Ung* deficiency over time. Nonetheless, using a highly MSH2‐dependent mechanism, *Ung^−/−^Smug1^−/−^* mice can still produce detectable levels of switched isotypes, especially IgG1. While not affecting the pattern of base substitutions, SMUG1 deficiency in an *Ung^−/−^* background further reduces somatic hypermutation at A:T base pairs. Our data reveal an essential requirement for uracil excision in class switching and in facilitating noncanonical mismatch repair for the A:T phase of hypermutation presumably by creating nicks near the U:G lesion recognized by MSH2.

## Introduction

In B cells, functional immunoglobulin genes are generated by gene rearrangement (V–D–J joining), giving rise to a primary repertoire of B cells producing antibodies of moderate specificity and affinity for many potential antigens. Upon antigen encounter, cells from this primary repertoire undergo further diversification in man and mouse by a process of somatic hypermutation (SHM) in which successive rounds of nontemplated nucleotide substitutions in the IgV gene are linked with antigen‐mediated selection to drive antibody affinity maturation, resulting in the production of antibodies with higher affinity. In addition, antigen encounter also leads to a shift in antibody isotype (from IgM to IgG3, IgG1, IgG2b, IgG2a, IgE, or IgA in the mouse, and analogously in other species) to change the antibody effector activity. All processes of postrearrangement antibody diversification (IgV SHM, IgC class switch recombination (CSR), and IgV gene conversion, which is not observed in mice and humans) are dependent on the activity of the enzyme AID, which acts by deaminating the DNA base cytosine (C) to uracil (U) in different regions of the immunoglobulin locus 1.

The initiating U:G lesion is recognized either owing to the fact that it constitutes a base mismatch (implicating the MSH2/MSH6 mismatch recognition heterodimer 4) or by virtue of the fact that uracil is an inappropriate base in DNA and therefore a target for base excision repair (BER) by uracil‐DNA glysosylases. Several such enzymes that have the ability to excise uracil from DNA have been described in mammalian cells (UNG; SMUG1; MBD4; TDG; 13), among which UNG appears to play the dominant role in class switching, since the efficiency of the process is reduced severalfold in UNG‐deficient mouse 16, human 17 and chicken cells 18. Nevertheless, substantial diversification still occurs in the absence of UNG, with UNG‐deficient mice showing normal levels of IgG1 in their serum despite very inefficient switching in vitro, pointing at the existence of a second pathway. Previous results from our group have revealed that the alternative CSR pathway can be essentially abolished by removal of MSH2 19, and others have shown similar effects for MSH6 deficiency 12, leading us to propose that direct recognition of the U:G lesion by MSH2/MSH6 mediated a glycosylase‐independent backup pathway 3. Although SMUG1, when heavily overexpressed, was able to catalyze class switching in *Ung^−/−^Msh2^−/−^* mice, the low endogenous levels of SMUG1 were seemingly insufficient to do so, and the lack of effect of enforced overexpression of SMUG1 in UNG‐deficient mice prompted speculation that SMUG1 might preferentially initiate error‐free repair at recognized lesions 19.

In SHM, recognition of the U:G mismatch by MSH2 results in recruitment of the translesion synthesis pathway, leading to resection and mutagenic DNA synthesis by polymerase η (Polη), which is largely responsible for the mutations at A:T pairs 9. In the absence of Polη, alternative translesion polymerases such as Polκ can contribute to this mutagenic mismatch repair (MMR) and give rise to mutations at A:T pairs 22. In the absence of MSH2, however, alternative translesion synthesis polymerases do not seem to support A:T mutagenesis with the recruitment of Polη being absolutely dependent on UNG, leading to the suggestion that UNG provides a backup to MSH2 for the recruitment of Polη during SHM 22.

The picture becomes more complicated when trying to reconcile the mechanistic insights gained from mouse models with data from human patients with immunoglobulin diversification pathologies, particularly when looking in detail at class switching to different immunoglobulin isotypes. Patients with deficiency in PMS2, the endonuclease immediately downstream of mismatch recognition by the MSH2/MSH6 heterodimer and absolutely necessary for conventional MMR, showed low serum levels of IgG2 and IgG4 and impaired CSR in vitro 27 with no effect on the pattern of SHM. However, *Pms2^−/−^* mice have a class switching deficit to IgG1 in vitro, while switching to IgG3 is hardly affected, but CSR in these mice is very dependent on the microhomology end‐joining pathway 28.

With regard to the role of UNG, the very rare (three so far) human patients again recapitulate the in vitro B‐cell phenotype of *Ung^−/−^* mice, but additionally show severe deficiency of switched isotypes in serum, which is less apparent (and for IgG1, not the case) in mice. Patients with intrinsic class switch deficit in vitro, often accompanied with residual IgG or IgA levels, have been identified in association with preferential use of microhomology at switch junctions, radiosensitivity, and tumor predisposition, hinting at an as yet unidentified DNA repair deficiency 29.

Thus, it appears that the subtle redundancies in the mechanistic pathways (MMR and BER) that promote both class switching and SHM have distinct effects on the different Ig subclasses in vivo both in mice and in human patients. Here, we test the contribution of endogenous SMUG1 to antibody diversification using *Smug1^−/−^* mice alone and in combination with *Ung^−/−^* mice in order to clarify the contribution of uracil excision to the secondary pathways of class switching and SHM.

## Results

### SMUG1 deficiency does not impair CSR in the presence of UNG

**Figure 1:**
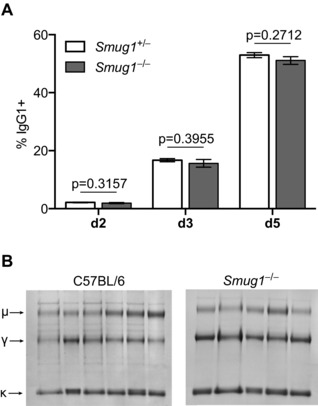
SMUG1 deficiency on its own has no effect on immunoglobulin CSR. (A) The proportion of B cells expressing surface IgG1 from *Smug1^−/−^* (gray bars) and littermate *Smug1^+/−^* (open bars) mice was monitored by flow cytometry over 5 days of culture after in vitro stimulation with LPS and IL‐4. Data show mean ± SEM from eight mice representative of three independent experiments (two‐tailed unpaired *t*‐test). (B) Serum immunoglobulin IgM and IgG proteins were purified on protein L and separated by SDS/PAGE, using *n* = 6 control mice and *n* = 5 *Smug1^−/−^* mice at 6 months of age. Arrows indicate the mobility of κ, kappa light chains, γ, IgG, and μ, IgM heavy chains.

Based on its biochemical activity on double‐stranded DNA, SMUG1 is assumed to be the uracil glycosylase dealing with the products of deaminated cytosine in mammals 30. At the immunoglobulin locus, UNG appears to be the main glycosylase accessing the enzymatically generated uracil, and it has been suggested that the fate of the U:G lesion might depend on competition between SMUG1 and UNG. Di Noia 21 and others 31 have put forward the idea of competition between faithful repair and mutagenesis, specifically that recognition by SMUG1 would lead to faithful repair of a double‐stranded substrate, whereas recognition by UNG during S phase of the cell cycle could create exposed breaks in single‐stranded regions of the replicating genome and promote class switching. It was therefore interesting to ascertain whether in the absence of SMUG1, accumulation of unrepaired uracil would lead to enhanced CSR, particularly in light of the observation that mutagenesis at the Ig locus by UNG is restricted to the G1 phase where it would have to compete with SMUG1 32. That is not the case, however, as in vitro class switching induced by LPS and IL‐4 in B cells from *Smug1^−/−^* mice 34 was indistinguishable from that observed in SMUG1‐proficient B cells and proceeded with the same kinetics (Fig. 1A). Equally, neither switching to IgG3 nor IgG2b was impaired in the absence of SMUG1 (not shown). This suggests that the accumulation of uracils induced by AID is not limited by the activity of SMUG1. Consistent with this, and in contrast to UNG‐deficient mice, *Smug1^−/−^* mice do not accumulate IgM in their serum as evidenced by the ratios of μ heavy chains versus γ chains purified by immunoprecipitation of kappa light chains by protein L (Fig. 1B).

### SMUG1 deficiency on an *Ung*^−/−^ background reduces serum IgG and IgA

To find out whether SMUG1 was responsible for the residual isotype switching in *Ung^−/−^* mice, we established a line of *Ung^−/−^ Smug1^−/−^* double‐knockouts. The *Smug1* gene ablation in these mice has previously been shown to lead to loss of the *Smug1* transcript and loss of biochemically detectable backup uracil glycosylase activity in nonlymphoid cells 34; we confirmed that this also applies to activated B lymphocytes (Supporting Information Fig. 1).

**Figure 2:**
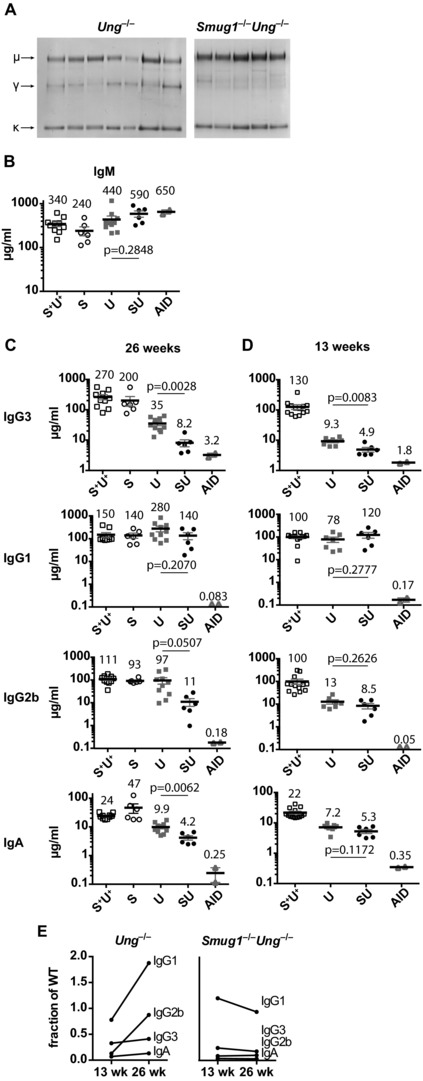
The age‐dependent partial compensation of CSR in UNG‐deficient mice is compromised in SMUG1/UNG double‐deficient mice. (A) Serum from 6‐month‐old *Smug1^−/−^Ung^−/−^* mice (*n* = 5) and *Ung^−/−^* mice (*n* = 7) was assessed for the presence of immunoglobulin chains by immunoprecipitation on protein L and gel electrophoresis. Arrows indicate the mobility of κ, kappa light chains, γ, IgG, and μ, IgM heavy chains (B, C). Immunoelectrochemiluminescent quantification of (B) IgM and (C) IgG3, IgG1, IgG2b, and IgA (in μg/mL) in serum collected at 26 weeks from mice of different uracil excision genotypes. Each symbol represents one mouse, with the mean (horizontal bars ± SEM) indicated above each group. Labels denote genotype: S^+^U^+^: *Smug1^+^ Ung^+^* control mice; S: *Smug1^−/−^*; U: *Ung^−/−^*; SU: *Smug1^−/−^Ung^−/−^*; AID: *Aicda^−/−^*. (D) Switched isotype titers were measured as in (C), using serum from mice at 13 weeks of age. (E) Changes in average titers of switched serum isotypes over time in *Ung^−/−^* versus *Smug1^−/−^Ung^−/−^*. Values taken from (C) and (D) are expressed as a fraction of the mean titers in uracil excision‐proficient mice. (C and D: Two‐tailed unpaired *t*‐test was used to compare *Ung^−/^*^−^ and *Smug1^−/−^Ung^−/−^* mice).

Analysis of serum immunoglobulin levels in *Ung^−/−^* mice confirmed and extended what we and others have seen previously 16, namely that UNG deficiency reduces the abundance of switched isotypes, with a marked accumulation of IgM that is even more pronounced in mice also lacking SMUG1 (Fig. 2A). Quantitation of serum immunoglobulin at 6 months by electrochemilumescent immunoassay confirmed that SMUG1 deficiency on its own does not increase the levels of IgM (Fig. 2B), nor does it affect the accumulation of switched isotypes (although as in the case of UNG deficiency, individual titers are quite scattered) (Fig. 2C). While *Ung^−/−^* mice show a reduction in serum IgG3 (from 270 to 35 μg/mL), IgG2b (from 111 to 97 μg/mL), and IgA (from 24 to 9.9 μg/mL), serum IgG1 levels are not diminished (mean value of 150 μg/mL in WTs compared with 280 μg/mL in *Ung^−/−^* mice). Quantitative analyses of serum from 26‐week‐old *Ung^−/−^ Smug1^−/−^* mice reveal that SMUG1 is necessary for most of the residual switching to IgG3, IgG2b, and IgA in UNG‐deficient mice, with loss of SMUG1 in this background causing a further reduction of IgG3 titers from 35 to 8.2 μg/mL, IgG2b from 97 to 11 μg/mL, and IgA from 9.9 to 4.2 μg/mL (Fig. 2C). Serum levels of IgG1 again are little affected by deficiency in both UNG and SMUG, although the hyper‐IgM phenotype is readily apparent in the double‐knockout (Fig. 2B).

To see if differences in the kinetics of SMUG1‐dependent switching had allowed mice to compensate for UNG deficiency over time, we repeated the serum immunoglobulin analyses on younger, 3‐month‐old mice. We reasoned that if switching by the secondary pathway was dramatically less efficient, it should only give rise to switched cells very occasionally, but once switched these cells could contribute to serum Ig levels for a considerable time, due to cell proliferation, life time, and the half‐life of Ig protein. Thus, an intrinsically inefficient class switching mechanism would be expected to be able to compensate appreciably only over time. We found that at 3 months (Fig. 2D), SMUG1 does contribute to the residual switching to IgG3, IgG2b, and IgA in UNG‐deficient mice (with loss of SMUG1 in this background causing a further reduction of IgG3 titers from 9.3 to 4.9 μg/mL, IgG2b from 13 to 8.5 μg/mL, and IgA from 7.2 to 5.3 μg/mL); however, this is a rather modest depression compared with the effect at 6 months. Nevertheless, the accumulation of IgM is already apparent in both *Ung^−/−^* and *Ung^−/−^Smug1^−/−^* genotypes (average 380 and 280 μg/mL, respectively, versus 180 μg/mL in controls, Supporting Information Fig. 2) and, again, IgG1 serum titers seem to be little affected by loss of UNG or of both UNG and SMUG1. We do note that there is still a small degree of switching to all isotypes in the absence of both glycosylases, relative to AID‐deficient mice.

Thus, loss of SMUG1 removes much of the residual switching to IgG2b, IgG3, and IgA in UNG‐deficient mice, and the age‐correlated partial restoration (Fig. 2E) of switched isotypes in the serum of UNG‐deficient but not SMUG1/UNG double‐deficient mice shows that the SMUG1‐dependent pathway does partially compensate for UNG deficiency.

### SMUG1 deficiency reduces the residual class switching to IgG1 in *Ung*^−/−^ mice in vitro

**Figure 3:**
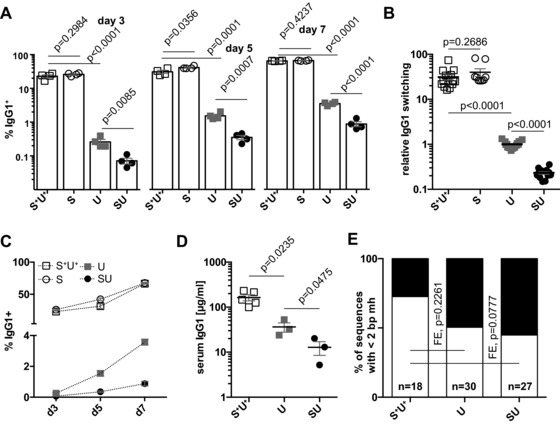
CSR in vitro is further impaired in uracil excision null B cells. (A) The proportion of IgG1^+^ B cells was determined by surface staining and flow cytometry after 3, 5, and 7 days of culture in vitro in the presence of LPS + IL‐4. Horizontal bars show mean ± SEM, with the value from cultures from individual animals represented by a symbol. Data shown are representative of six independent experiments. (B) Splenic B cells were cultured for 5 days and the fraction of IgG1^+^ cells was determined as in (A) and expressed as a fraction of the per‐experiment average obtained from UNG‐deficient animals. Data aggregated from six independent experiments comprising a total of 50 individual animals indicated as data points. (C) Using data from (A), genotype‐dependent changes were assessed in the fraction of switched cells in culture at the three indicated time points. (D) Serum IgG1 titers (μg/mL) at 6 weeks of age were assessed by ELISA from litters born to *Aicda^−/−^* mothers and deficient in both UNG and SMUG1 or UNG only and compared with uracil excision‐proficient controls. Each symbol represents the titer of an individual mouse with the means shown as horizontal bars ± SEM. Data are from three independent litters. (E) Sequence homology in Sμ–Sγ1 junctions PCR‐amplified from splenic B cells after 7 days of culture in LPS and IL‐4. In black, the proportion of junctions with two or more residues identical to both μ and γ1 switch regions. *n* indicates the number of sequences analyzed per genotype. Labels denote genotype: S^+^U^+^: *Smug1^+^ Ung^+^* control animals; S: *Smug1^−/−^*; U: *Ung^−/−^*; SU: *Smug1^−/−^Ung*. (A, B, and D: two‐tailed unpaired *t*‐test; E: Fisher's exact test).

The finding that serum IgG1 levels are scarcely affected by glycosylase deficiency in mice, although consistent with previous observations from us and others, is in stark contrast to IgG1 switching observed in vitro by culturing splenic B cells in the presence of LPS + IL‐4. Analysis of such cultures using B cells from WT, *Ung^−/−^, Smug1^−/−^* and *Ung^−/−^Smug1^−/−^* mice over 7 days of culture confirms that UNG deficiency does indeed reduce switching to IgG1 in this assay severalfold, as reported previously. In addition, we now find that the residual switching observed in UNG‐deficient mice is reduced a further severalfold (though not entirely ablated) if the cells are also deficient in SMUG1 (Fig. 3A–C). Similar effects are found on the residual in vitro switching to other isotypes (Supporting Information Fig. 3). Thus, the picture emerges that SMUG1 is a major contributor to UNG‐independent switching.

### SMUG1 deficiency reduces the residual class switching to IgG1 in *Ung*^−/−^ mice in vivo

In view of our findings that SMUG1‐proficient *Ung^−/−^* mice accumulate greater levels of some switched isotypes between 3 and 6 months of age compared with SMUG1‐deficient *Ung^−/−^* mice, and given the strong effect of SMUG1 deficiency on switching in vitro, we were puzzled by the lack of effect observed in vivo with respect to IgG1.

This could either mean that switching to IgG1 proceeded via an entirely different mechanism in vivo, or that while still uracil glycosylase‐dependent, serum accumulation occurred with faster kinetics so that the effects were masked at 3 months. To discriminate between the two alternatives, we tested serum IgG1 levels in 6‐week‐old *Ung^−/−^* and *Ung^−/−^Smug1^−/−^* littermates born to AID‐deficient mothers (to exclude maternal transmission of switched isotypes). The results reveal that indeed switching to IgG1 in vivo is greatly reduced in the absence of uracil excision (13 μg/mL compared with 160 μg/mL in controls) and that SMUG1 can provide a significant back up activity (from 13 μg/mL in the double‐deficient to 36 μg/mL in solely UNG‐deficient mice, Fig. 3D), well above the background levels observed in even older *Aid^−/−^* mice (<0.2 μg/mL).

We have previously shown that simultaneous deficiency in both UNG and MSH2 causes a near total block on switching to IgG as judged by both in vitro switching to IgG1 in LPS + IL‐4 cultures as well as in vivo by SDS/PAGE analysis of the protein L‐binding serum immunoglobulin fraction 19. We suspect our new findings reflect that both UNG‐ and SMUG1‐initiated switching can be facilitated by the downstream action of MSH2, with the SMUG1‐pathway being especially sensitive to loss of MSH2 (whereas UNG can lead to robust MSH2‐independent switching). Indeed, given its low abundance 19, biochemical activity (compared with UNG, Supporting Information Fig. S1), and particularly low activity on AID hotspot motifs 36, it is likely that SMUG1 may excise fewer uracils than UNG during the switching process, resulting in an increase in the average distance between abasic sites (and consequently nicks) on the two DNA strands. Consistent with this model, we observe a tendency for the switched γ1 junctions obtained in vitro from *Ung^−/−^ Smug1^−/−^* B cells to show greater junctional homology than is observed in WT controls (Fig. 3E).

### SMUG1 deficiency reduces mutation accumulation at A:T pairs during hypermutation in Ung^−/−^ mice

**Figure 4:**
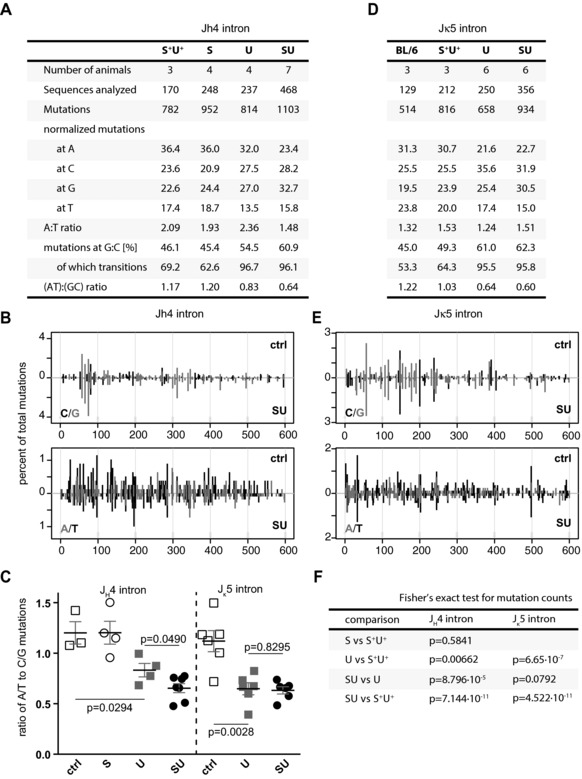
SHM analysis of germinal center B cells reveals a dependence of A:T hypermutation on uracil excision. (A) Mutations observed in the J_H_4 intronic region from rearranged Peyer's patches B cells. The total number of mutations from the indicated individual animals is shown for each genotype. The mutations at each base, expressed as a percent of the total, were normalized to the base composition of the region analyzed. (B) Distribution of nucleotide substitutions at G:C and A:T pairs in the J_H_4 intron. Comparison of uracil excision‐proficient (control, top histogram) versus uracil excision‐deficient (*Smug1^−/−^Ung^−/−^*, bottom histogram) B cells. Each bar represents percent of total mutations. (C) The normalized ratios of A:T to C:G mutations in datasets from each individual mouse are shown grouped per genotype. Each symbol represents the value obtained for an individual mouse with bars indicating the mean ± SEM (two‐tailed unpaired *t*‐test). (D, E) The J_κ_5 intronic region was analyzed as in (A) and (B). (F) Statistical comparison of mutation distributions using Fisher's exact test. Labels denote genotype: S^+^U^+^ and BL/6: *Smug1^+^ Ung*^+^ and C57BL/6J controls (ctrl), respectively; S: *Smug1^−/−^*; U: *Ung^−/−^*; SU, *Smug1^−/−^Ung^−/−^*.

The findings that endogenous SMUG1 does access the immunoglobulin switch regions and provide a backup for UNG led us to ask whether deficiency in SMUG1 also impacted SHM of the IgV gene. Sequence analysis of the rearranged IgV_H_ 3′‐flanking region from *Smug1^−/−^, Ung^−/−^*, and *Ung^−/−^ Smug1^−/−^* double‐knockout mice revealed that whereas UNG deficiency causes a substantial skewing in the nucleotide substitution pattern (abolishing transversions at C:G pairs), deficiency in SMUG1, whether in an UNG‐proficient or ‐deficient background, causes no major additional perturbation in the pattern or distribution of nucleotide substitutions (Fig. 4A and B). Detailed analysis, however, reveals that SMUG1 deficiency in an *Ung^−/−^* background causes a further perturbation of the distribution of mutations across the four bases (Fisher's exact test for mutation counts: *Smug1^−/−^ Ung^−/−^* versus *Ung^−/−^*, *p* = 0.00008796), which we attribute largely to a reduction in the mutation frequency at A:T pairs. This is reflected in the decreased ratio of mutations at A:T base pairs relative to C:G pairs, which goes beyond that attributable to UNG deficiency alone (Fig. 4C and Supporting Information Fig. 4). A similar analysis of the Ig_κ_ light chain locus, focusing on the J_κ_5 3′‐flanking region of productively rearranged V_κ_ segments, yielded broadly similar conclusions (Fig. 4D and E), namely a reduction of mutations at As and Ts in the absence of uracil excision. The contribution of SMUG1 in an *Ung^−/−^* background was not apparent in the kappa locus (Fisher's exact test *Smug1^−/−^ Ung^−/−^* versus *Ung^−/−^*, *p* = 0.09784), likely due to the overall lower mutation accumulation. A summary of the statistical comparison of mutation distributions between all four genotypes is presented in Figure 4F.

## Discussion

**Figure 5:**
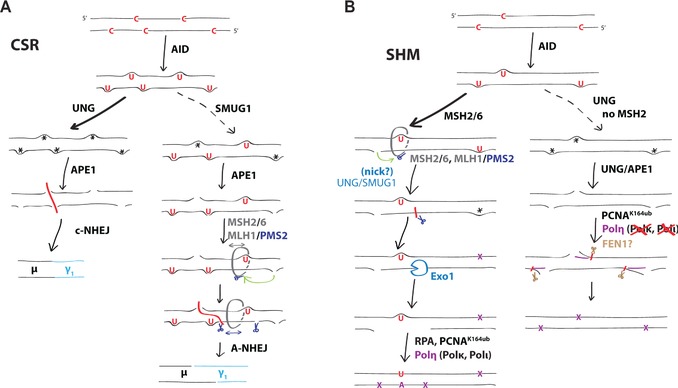
Uracil recognition pathways in antibody diversification. (A) CSR. Efficient base excision by UNG (or by highly overexpressed SMUG1) of densely spaced uracil residues in the immunoglobulin switch region repeats followed by cleavage by APE1 results in frequent double‐strands breaks that can be joined by nonhomologous end joining with minimal end processing (left path). In the absence of UNG, or in regions with lower AID hotspot density (right path), nicks are typically too far apart to allow direct formation of double‐strand breaks. Class switching thus relies on processing of U:Gs by the MMR machinery (comprising the MSH2/MSH6//MLH1/PMS2 heterotetramer) to create staggered breaks, which are resolved via microhomology‐dependent alternative end joining. Even rare nicks, mostly provided by SMUG1 in the absence of UNG, could facilitate Exo1 resection by licensing of the endonuclease activity of PMS2 in the MMR complex, and thus promote the formation of double‐strand breaks leading to class switching. (B) SHM. The contribution of uracil excision to the MHS2‐dependent phase 2 (left path) is evidenced by the lowered proportion of A:T mutations observed in the absence of UNG, which is further depressed in the absence of SMUG1. The creation of an MMR‐dependent resection patch is known to be potentiated by nicks in the region surrounding the mismatch, suggesting that uracil excision facilitates phase II by providing a source of nicks. As in the case of CSR, even isolated, distal nicks could license the PMS2 endonuclease to make further incisions and promote efficient patch resection. It is unknown, but plausible, that mutagenic MMR in the absence of uracil excision by both UNG and SMUG1 exploits other sources of nicks. Subsequent to Exo1 resection, the patch is refilled by error‐prone Y‐family polymerases, among which Polη plays the dominant role for hypermutation. In the absence of MSH2 (right path), efficient uracil excision by UNG is absolutely essential for mutagenesis at A:T pairs, and Polη cannot be substituted for by other endogenous polymerases, suggesting that they cannot (with detectable efficiency) facilitate mutagenesis across the relatively short patches of BSR.

We have previously proposed that there are two main pathways of class switching — one (the major pathway) initiated by UNG recognition of the U:G lesion with its efficiency dependent on a downstream role of MSH2, while we envisaged the other (minor) pathway to be initiated by MSH2 recognition of the U:G mismatch and also resolved by the MMR pathway 3. Curiously, while enforced overexpression of hSMUG1 in an *Ung^−/−^ Msh2^−/−^* background resulted in CSR above that observed in UNG‐deficient mice, overexpression in *Ung^−/−^ Msh2^+/+^* mice did not further increase class switching 21, suggesting that SMUG1 operated independently of and possibly competed with the MSH2‐dependent pathway. The results presented here clearly show that endogenous SMUG1 provides sufficient uracil glycosylase activity in the relevant B‐cell population and is required for efficient MSH2‐dependent switching in the absence of UNG, placing uracil BER upstream of the MMR pathway (Fig. 5).

However, in contrast to UNG/MSH2 double‐knockouts, some level of switching remains in the SMUG/UNG mice, particularly when looking at serum levels of IgG1, which are only affected by deficiency in UNG and/or SMUG1 very early in life. Thus, the MMR pathway can promote CSR independent of the uracil glycosylases UNG and SMUG1, either via a true MMR‐only pathway, possibly exploiting low levels of endogenous or induced nicks 37 or by relying on very low levels of uracil excision activity provided by further backup glycosylases that are not readily detectable in biochemical assays 5.

We think the most plausible model (see Fig. 5A) to explain the particular MSH2 dependence of SMUG1‐initiated class switching is that the role of MSH2 in downstream processing becomes especially apparent when the uracil excision does not initially generate double‐strand breaks because, for example, the uracil base excision events are quite rare and distantly spaced and thus require further processing (i.e. resection): this would account for the slight shift toward microhomology‐mediated switching in these mice, analogous to what has been observed in switch junctions from human UNG‐deficient patients 40, consistent with the proposal of Stavnezer and colleagues 1. This model could also explain why the IgG1 isotype, having the largest contiguous repetitive switch region that like all switch repeats has an abundance of AID hotspots (see Ensembl GRCm38 genome build), might be most easily switched to in the absence of UNG, with a very high density of lesions making up for SMUG1's relative inefficiency.

Several curious anomalies come out of this work. Deficiency in UNG and SMUG1 affects all switched isotypes studied, but to different extents. In particular, there is a striking contrast between switching to IgG1 as monitored in in vitro cultures (where an effect of UNG and SMUG1 deficiency is readily discerned) from what is observed when serum accumulation is examined (where the MSH2‐dependent pathway quickly and fully compensates for SMUG1/UNG double deficiency). Also curiously, it is known that human patients with UNG deficiency have very low serum IgG levels, much lower than those observed in UNG deficient or even what we now see in (UNG + SMUG1)‐deficient mice.

All these findings could in principle be reflecting differential contributions of the switching pathways at different developmental or immunological stages; in particular, the effects of SMUG1 deficiency are more apparent with age, as the relative inefficiency of the SMUG1 pathway is only able to compensate for UNG deficiency over time. Similarly, Di Noia and colleagues 35 have investigated the antibody response of *Ung^−/−^* mice and found that while total serum IgG1 levels were unaltered, production of switched isotypes upon antigen encounter was markedly delayed in vaccination models.

Our data from *Smug1^−/−^ Ung*^−/−^ and *Ung^−/−^* mice born to AID‐deficient mothers clearly show that in vivo such B cells do rely on SMUG1, suggesting that the deficiency of CSR in vitro truly reflects that expected in an acute immune response and suggesting that even a small starting population of switched cells can accumulate and expand in vivo to normal levels but only over time. More importantly, this also reveals that uracil excision is still the main pathway for class switching to all isotypes, including IgG1 in vivo.

Finally, the difference that emerges from a comparison between the data from mouse and humans could reflect a species difference in the biochemical activity of SMUG1 42 and/or the operation of the MSH2‐initiated pathway. However, it could be also very well that the few UNG‐deficient patients analyzed so far represent a clinical ascertainment bias as they were identified on the basis of their immunological phenotype.

With regard to SHM, our results suggest that, in the absence of UNG, SMUG1 only rarely or inefficiently accesses the initiating U:G lesion before it is either replicated over (to give C‐>T and G‐>A transitions) or recognized by MSH2 (leading to phase 2 mutations largely at A:T pairs), so that the pattern of nucleotide substitutions is not substantially perturbed. Nonetheless, removal of SMUG1 in an UNG‐deficient background results in a small, but convincing, diminution in the proportion of mutations at A:T pairs. This would fit well with a model where a glycosylase‐provided nick, possibly away from the primary U:G lesion, facilitates entry and/or licensing of the MMR machinery to initiate long‐patch resection for mutagenesis at A:T pairs (Fig. 5B). This model would also account for the known lack of direct association between C:G mutation hotspots and A:T mutations and our observation that transversion mutations at C:G pairs are not further diminished in the UNG/SMUG1 double‐knockout below the levels observed in the UNG knockout. Inefficient uracil excision (e.g. at 1–2% of Us) might not be detectable as transversions in the SHM assay, but could nevertheless lead to significant switching, which may require excision of only a small proportion of U:G mispairs.

Whatever the explanation, the reduced mutation at A:T pairs in the absence of both UNG and SMUG1 support the idea that the catalytic activity of the glycosylase, more than its structural properties, facilitates recruitment of the protein factors that matter for hypermutation, and similarly (but more robustly) for CSR.

Our data reveal that uracil excision by endogenous SMUG1 contributes to antibody diversification in the absence of UNG, and place uracil excision upstream of the MSH2 pathway. The extent of SMUG1's contribution (always much less than that of UNG) differs according to the readout measured but it is clear that class switching strongly depends on SMUG1 in the absence of UNG. Inefficient class switching still occurs in the absence of both glycosylases, presumably triggered by MSH2 recognition of the initiating lesion and exploiting other sources of nicks.

With regard to SHM, we show that the amount of SMUG1 activity in a UNG‐deficient hypermutating cell is not sufficient to excise uracil from many of the AID‐generated U:G lesions before the replication fork passes but reveal that even a small contribution from UNG or SMUG1 to uracil excision (while not providing the sole route of entry into phase 2) impacts on the efficiency of mutations at A:Ts. An attractive possibility is that glycosylase‐catalyzed uracil excision at another U:G lesion licenses the MSH2/PMS2 complexes to promote an efficient mutagenic repair.

## Materials and methods

### Mice

The generation of *Smug1*^−/−^ mice and their intercrossing have been described previously 34. *Ung*^−/−^ mice 43, *Msh2*^−/−^ mice 44, and *Aicda^−/−^* mice 45 have been described before. Mice used for analysis of switched isotype serum levels in the absence of maternal immunoglobulins were produced by crossing *Aicda^−/−^ Ung*^−/+^ *Smug1*^−/+^ females with *Aicda^+/+^ Ung*^−/+^ *Smug1*^−/+^ males. Animal studies were performed under the EU directives and UK Home Office Project Licence 70/7571 with consent of the LMB Animal Welfare and Ethical Review Body.

### In vitro CSR assay

This assay was performed as described previously 21, with modifications. Briefly, resting naïve B cells were isolated from splenocyte suspensions after erythrocyte lysis with ammonium chloride via depletion of CD43^+^ cells using anti‐CD43 microbeads and LD columns (Miltenyi Biotec). Cells were cultured in RPMI1640 with 10% FBS (Hyclone), 1× penicillin/streptomycin (Gibco), and 50 μM β‐mercaptoethanol. To induce CSR to IgG1, cells were cultured in the presence of 40 μg/mL LPS (Sigma L4391), 25 ng/mL recombinant murine (rm) IL‐4 (R&D Systems, 404‐ML‐010), and 20 ng/mL rmBAFF (R&D Systems, 2106‐BF‐010). CSR to IgG3 was induced by 40 μg/mL LPS, to IgG2b by a combination of LPS, BAFF, and 2 ng/mL rmTGF‐β1 (Cell Signaling Technology), and CSR to IgA was induced by culturing with LPS, IL‐4, BAFF, TGF‐β1, and 1.5 ng/mL rmIL‐5 (eBioscience), as described in 46.

### Flow cytometric analysis

Cells were harvested, washed with PBS, stained with eF780‐conjugated viability dye (eBioscience), and Fc receptors blocked using 2% rat serum in PBS. Surface staining for IgG1 was performed using FITC‐conjugated rat anti‐mouse IgG1 (clone A85–1, Becton Dickinson) in the presence of 1% rat serum. For class switching to IgG3, IgG2b, and IgA, cells were fixed and permeabilized using the Cytofix/Cytoperm kit (Becton Dickinson), stained intracellularly with biotinylated anti‐IgG3 (clone R40–82, Becton Dickinson), biotinylated anti‐IgG2b (clone R12–3, Becton Dickinson) or biotinylated anti‐IgA (clone C10–1, Becton Dickinson), and Streptavidin‐allophycocyanin (Becton Dickinson). Switching was calculated as the percentage of FITC or allophycocyanin positive singlet viable cells in the lymphocyte gate.

### Serum immunoglobulin immunoprecipitation

IPs were performed essentially as described previously 19. Briefly, 20 μL protein L‐conjugated sepharose beads (BioVision) were blocked in 500 μL 2% glycine/PBS, washed in PBS, and then 5 μL of serum diluted to 500 μL in PBS were added and rotated for 1 h at room temperature. Beads were washed thrice in PBS/0.05% Tween‐20, then boiled in 1× SDS loading buffer (Life Technologies) supplemented with β‐mercaptoethanol and separated on 4–12% SDS/PAGE gels (Life Technologies) before Coomassie staining (InstantBlue, Expedeon).

### Serum immunoglobulin quantification

Analysis of mouse immunoglobulin isotypes in serum was performed using a multiplexed immunoelectrochemiluminescent mouse isotyping kit (Meso Scale Discovery, Gaithersburg, MD, USA) according to the manufacturer's instructions with exclusion of IgG_2a_ results as there was significant (∼0.9%) cross‐reactivity with IgM (data not shown). For IgG1 in mice from AID‐deficient mothers, we performed a conventional ELISA using A85–3 as capture and biotinylated A85–1 as detection antibody (both BD Biosciences). 10% FBS in PBS‐T (0.05% Tween‐20 in PBS) was used for blocking and PBS‐T for washing.

### Isolation of GC B cells and analysis of SHM

Germinal center B cells were isolated from Peyer's patches of mice ∼6 months of age, essentially as described 5. Cells were lysed for PCR using DirectPCR cell lysis reagent (Viagen Biotech) and the 3′ flanking intron of V_H_J558(D) Jh4 rearrangements amplified using KOD Hotstart DNA polymerase (Merck Millipore). J_κ_5 introns were amplified using degenerate primers 48. PCR product bands of the correct size were gel purified, digested with SpeI and EcoRI, and cloned into Bluescript II SK(+), then sequenced with M13‐FP. Three C57BL/6J mice were used as controls for the J_κ_5 intronic sequences in addition to littermate controls.

Sequence quality clipping and alignment was performed using GAP4 49 and clonally related sequences removed on the basis of CDR3 divergence of <3% if they shared mutations in the intronic region.

### Microhomology analysis of immunoglobulin switch junctions

Cultured splenocytes were lysed and switch junctions amplified from diluted template as described 28. PCR products were cloned into pCR‐Blunt using the ZeroBlunt kit (Invitrogen) and sequenced using M13 primers. Switch junctions were scored according to the extent of perfect homology.

## Conflict of interest

The authors declare no financial or commercial conflict of interest.

**Abbreviations**
BER: base excision repair
CSR: class switch recombination
MMR: mismatch repair
Polη: polymerase η
SHM: somatic hypermutation

## Supplementary Material

As a service to our authors and readers, this journal provides supporting information supplied by the authors. Such materials are peer reviewed and may be re‐organized for online delivery, but are not copy‐edited or typeset. Technical support issues arising from supporting information (other than missing files) should be addressed to the authors.

**Figure S1**. Uracil Glycosylase activity of in‐vitro‐activated B cell extracts.
**Figure S2**. Serum IgM titers in mice 13 weeks of age.
**Figure S3**. Class switching to IgG3 and IgG2b after 5 days of culture.
**Figure S4**. Frequency of nucleotide substitutions at A, C, G and T bases.

Peer review correspondence
