# Supplementary material for: Uracil excision by endogenous SMUG1 glycosylase promotes efficient Ig class switching and impacts on A:T substitutions during somatic mutation
Source: Eur J Immunol. 2014 May 27;44(7):1925–35. doi: 10.1002/eji.201444482 (PMC4158878; doi:10.1002/eji.201444482)
Supplement: Supplementary file 1 — Figure S1. Uracil Glycosylase activity of in‐vitro‐activated B cell extracts. Figure S2. Serum IgM titers in mice 13 weeks of age. Figure S3. Class switching to IgG3 and IgG2b after 5 days of culture. Figure S4. Frequency of nucleotide substitutions at A, C, G and T bases. [file eji-44-1925-s6.pdf]

European Journal of Immunology

Supporting Information
for
DOI 10.1002/eji.201444482

Felix A. Dingler, Kristin Kemmerich, Michael S. Neuberger and Cristina Rada

Uracil excision by endogenous SMUG1 glycosylase promotes efficient Ig class switching and impacts on A:T substitutions during somatic mutation

© 2014 The Authors. *European Journal of Immunology* published by WILEY-VCH Verlag GmbH & Co. KGaA, Weinheim

SUPPORTING INFORMATION

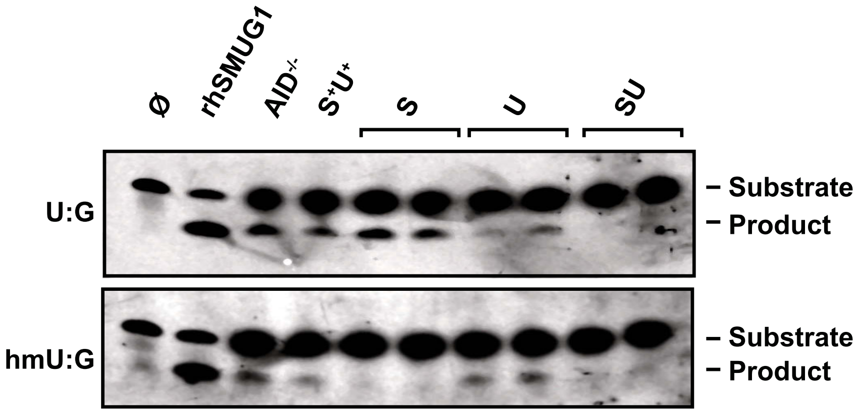

Supplementary Figure 1. Uracil Glycosylase activity of in-vitro-activated B cell extracts. Top panel: activity on mismatched uracil in the context of a double stranded oligonucleotide substrate. Bottom panel: activity on 5-hydroxymethyl-uracil, which is diagnostic for SMUG1 activity.

B cells cultured in the presence of LPS/IL-4/BAFF were collected at day 7 and whole cell extracts were used to assess uracil excision as described previously [34]. Briefly, equivalent cultures were washed in PBS, then lysed on ice in 10 mM Tris, pH=8.0, 150 mM NaCl, 1 mM EDTA, 1 mM DTT, 0.1 % Nonidet-P40, 20 % glycerol, and Complete protease inhibitor (Roche) for 1 hr with occasional mixing, debris removed by pelleting, and the supernatants dialyzed against reaction buffer. Equal volumes of dialysate were assayed in the presence of APE1 (2.5 U New England Biolabs). Dialysis buffer or 5 U of recombinant human SMUG1 (New England Biolabs) served as negative (ø) and positive controls (rhSMUG1), respectively. The genotype of the cells used is denoted above. S^+U^+: *Smug1*$^+$ *Ung*$^+$ control animals; S: *Smug1*$^{-/-}$; U: *Ung*$^{-/-}$; SU: *Smug1*$^{-/-}$*Ung*$^{-/-}$).

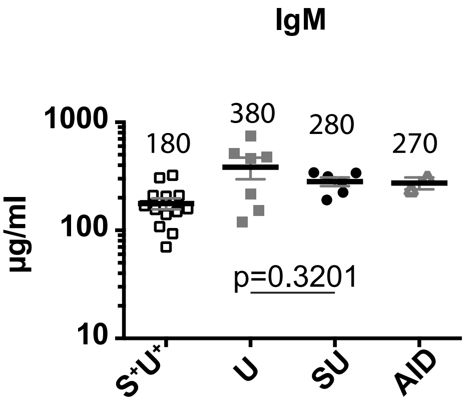

Supplementary Figure 2. Serum IgM titers in mice 13 weeks of age.

Titers were determined by immuno-electrochemiluminescence. Labels indicate genotypes as before. $S^{+}U^{+}$: *Smug1*$^{+}$ *Ung*$^{+}$ control animals; U: *Ung*$^{-/-}$; SU: *Smug1*$^{-/-}$*Ung*$^{-/-}$; AID: *Aicda*$^{-/-}$.

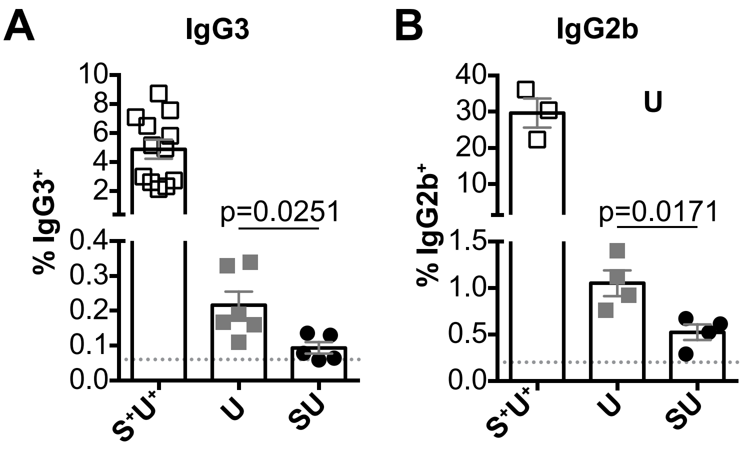

Supplementary Figure 3. Class switching to IgG3 and IgG2b after 5 days of culture.

The fraction of cells staining positive for intracellular IgG3 and IgG2b is shown with the dotted horizontal line representing the staining observed in AID-deficient or UNG/MSH2-deficient cells which in both cases were indistinguishable from background in this assay. Bars indicate mean ± SEM, with symbols indicating titers from individual animals. (Two-tailed unpaired *t*-test). Labels denote genotype, S^+U^+: *Smug1*$^+$ *Ung*$^+$ control animals; U: *Ung*$^{-/-}$; SU: *Smug1*$^{-/-}$*Ung*$^{-/-}$.

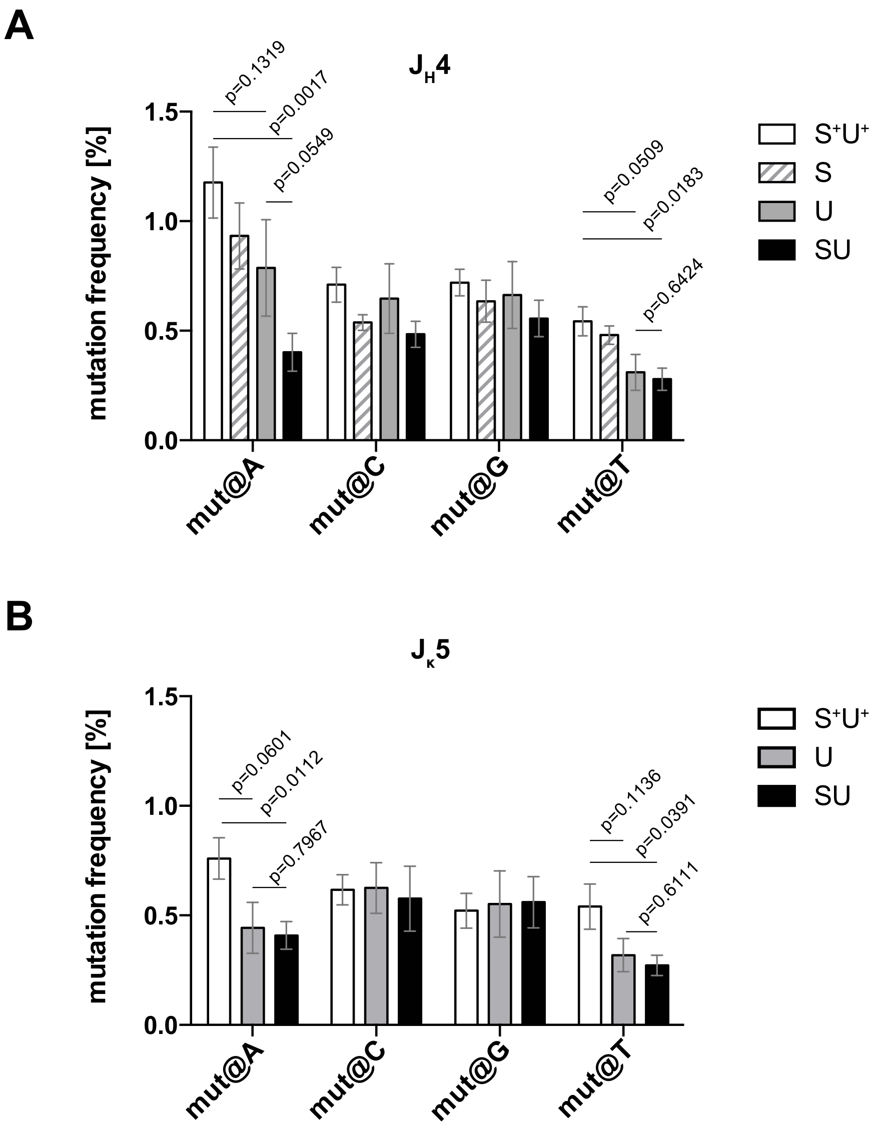

Supplementary Figure 4. Frequency of nucleotide substitutions at A, C, G and T bases. The frequency of mutations at each base is calculated as a percentage of the total number of each base in the reference sequence times the number of sequences analyzed in the J_H4 (A) and $J_\kappa5$ intron (B). Labels indicate genotypes as before. S^+U^+: *Smug1*$^+$ *Ung*$^+$ control animals; S: *Smug1*$^{-/-}$; U: *Ung*$^{-/-}$; SU: *Smug1*$^{-/-}$*Ung*$^{-/-}$. (Two-tailed unpaired *t*-test).
