# Supplementary material for: Uracil excision by endogenous SMUG1 glycosylase promotes efficient Ig class switching and impacts on A:T substitutions during somatic mutation
Source: Eur J Immunol. 2014 May 27;44(7):1925–35. doi: 10.1002/eji.201444482 (PMC4158878; doi:10.1002/eji.201444482)
Supplement: Supplementary file 2 — Peer review correspondence [file eji-44-1925-s7.pdf]

European Journal of Immunology

# Peer review correspondence

## Uracil excision by endogenous SMUG1 glycosylase promotes efficient Ig class switching and impacts A:T substitutions during somatic mutation

Felix A Dingler, Kristin Kemmerich, Michael S Neuberger and Cristina Rada

Correspondence: Dr. Cristina Rada, MRC Laboratory of Molecular Biology, Francis Crick Avenue, Cambridge CB2 0QH, U.K.

---

| | | |
|---|---|---|
| Review Timeline: | Submission date: | 17 January 2014 |
| | First Editorial decision: | 17 February 2014 |
| | First Revision received: | 6 March 2014 |
| | Accepted: | 15 April 2014 |

---

Handling Executive Committee member: Prof. Hans-Martin Jack

Please note that the correspondence below does not include the standard editorial instructions regarding preparation and submission of revised manuscripts, only the scientific revisions requested and addressed.

**<u>First Editorial Decision – 17 February 2014</u>**

Dear Dr. Rada,

Manuscript ID eji.201444482 entitled "Uracil excision by endogenous SMUG1 glycosylase contributes to efficient Ig class switching and impacts on A:T substitutions during somatic mutation" which you submitted to the European Journal of Immunology has been reviewed. The comments of the referees are included at the bottom of this letter.

A revised version of your manuscript that takes into account the comments of the referees will be reconsidered for publication. In particular, we strongly recommend that you address referee 3's concern regarding the use of a digestion-circularization PCR approach to confirm your results.

You should also pay close attention to the editorial comments included below. *In particular, please edit your figure legends to follow Journal standards as outlined in the editorial comments. Failure to do this will result in delays in the re-review process.*

Please note that submitting a revision of your manuscript does not guarantee eventual acceptance, and that your revision will be re-reviewed by the referees before a decision is rendered.

European Journal of Immunology

If the revision of the paper is expected to take more than three months, please inform the editorial office. Revisions taking longer than six months may be assessed by new referees to ensure the relevance and timeliness of the data.

Once again, thank you for submitting your manuscript to European Journal of Immunology and we look forward to receiving your revision.

Yours sincerely,
Karen Chu

On behalf of Prof. Hans-Martin Jack

Dr. Karen Chu
Editorial Office
European Journal of Immunology
e-mail: ejied@wiley.com
www.eji-journal.eu

*****************************************************

Reviewer: 1

Comments to the Author
It has long been a question if UNG is the only uracil glycosylase involved in class switch recombination and somatic hypermutation. There is residual switching in UNG-deficient mice. More importantly, A:T mutations, which depend on a nick for access by pol eta, are mostly unaffected in UNG-deficient mice. What is the nick due to? Here, Dingler et al. show that SMUG is a back-up glycosylase. Using mice doubly-deficient for UNG and SMUG, switching is reduced greater than UNG deficiency alone. This is important because although the authors previously found that overexpression of SMUG had an effect, it wasn't clear if endogenous SMUG was doing anything. Finally, the authors suggest that a potential source of nicks for pol eta in UNG-deficient cells could be due to SMUG-generated abasic sites, which are then nicked by APE1. However, B cells still retain about 40% A:T mutations in the absence of both glycosylases, suggesting that another source of nicks is predominant—perhaps Okazaki fragments.

European Journal of Immunology

Specific comments

Fig. 4 A and D. What are the mutation frequencies? What is the W:S ratio?

Fig. 4 C. What is the significance (p-value) of the drop in A:T mutations in JH4 between those found in Ung and Smug/Ung cells? The data for Jk5 looks like there is no difference, so it would not be “broadly similar” to the JH data (p. 11, line 15). Also on p. 14, line 15, “small but convincing” (assuming the statistics hold up) does not apply to the Jk5 data.

Fig. 4 D and E are not mentioned the text.

Reviewer: 2

Comments to the Author

This paper demonstrates that Smug1, a uracil glycosylase, contributes to both Ig class switch recombination (CSR) and somatic hypermutations (SHM) in B cells lacking UNG. It might make a small contribution even in UNG-proficient cells but it is much less important than UNG in excising dUs introduced by AID activity. The study is complete and the discussion of the literature and their own data are excellent. This is an important contribution about a complicated and somewhat controversial subject.

My criticisms are minor.

I think they should determine if CSR to IgG3 and IgG2b are decreased in the absence of Smug1. I think IgG1 is the wrong isotype. This is not terribly important in the context of the entire manuscript, which clearly shows that Smug1 has a small effect when UNG is missing, but it would have been interesting to see if Smug1 deficiency affected isotypes that are likely to be more dependent on the frequency of DNA breaks.

They should provide the frequency of SHM observed in the 4 different genotypes they study for the Jh4 and Jk5 introns.

They should also provide a reference for their statement that Sg1 is the most repetitive and hotspot-enriched switch region (p. 12). My information disagrees with this statement, although since Sg1 consists of both repetitive and non-repetitive sequences, it is possible that there is a calculation for hoptspot density that excludes the latter regions and this gives rise to their statement. This should be referenced.

Reviewer: 3

Comments to the Author

In both the title and abstract of this manuscript, the authors conclude that SMUG1 contributes to efficient Ig class switching. However, none of the data in the manuscript support this conclusion. The data do

suggest that SMUG1 might provide a low “backup” level of CSR in situations where UNG is absent. However, there is no statistical evaluation of the data supporting this “backup” role, so even this limited function has not been demonstrated. If the manuscript is revised to include the requisite statistical evaluation of the SMUG1 CSR function, it is still not clear that the demonstration of this “backup” CSR activity would be sufficiently important to a broad immunological audience to warrant publication in EJI.

It is also surprising that the authors did not use a digestion-circularization PCR approach to directly measure the production of CSR DNA joins in cells stimulated to switch in culture; instead relying on FACS measurement of the production of surface isotype-switched Ig proteins (which assumes that transcription, translation and surface deposition of protein provide a directly linear relationship with DNA recombination events).

The following figures require statistical analyses to show whether differences noted in the text between US and U mice are significant: Fig 2, Fig. 3, Fig. 4, Sup. Fig. 3.

The methodology for obtaining the mutant mice born from AID-/- mothers should be briefly described.

**First Revision – authors’ response – 6 March 2014**

Dingler et al. Point by point reply to Reviewers comments

Reviewer: 1

Comments to the Author

It has long been a question if UNG is the only uracil glycosylase involved in class switch recombination and somatic hypermutation. There is residual switching in UNG-deficient mice. More importantly, A:T mutations, which depend on a nick for access by pol eta, are mostly unaffected in UNG-deficient mice. What is the nick due to? Here, Dingler et al. show that SMUG is a back-up glycosylase. Using mice doubly-deficient for UNG and SMUG, switching is reduced greater than UNG deficiency alone. This is important because although the authors previously found that overexpression of SMUG had an effect, it wasn’t clear if endogenous SMUG was doing anything. Finally, the authors suggest that a potential source of nicks for pol eta in UNG-deficient cells could be due to SMUG-generated abasic sites, which are then nicked by APE1. However, B cells still retain about 40% A:T mutations in the absence of both glycosylases, suggesting that another source of nicks is predominant—perhaps Okazaki fragments.

We thank referee 1 for recognizing the importance of this work, and agree that Okazaki fragments could well constitute a source of nicks. We have not mentioned them specifically since the timing of their occurrence in the cell cycle seems at odds (although not irreconcilably so) with data from the Jolly and Raynaud labs who suggest antibody diversification is confined to the G1 phase of the cell cycle [Faili et al. Nature Immunology 2002, Sharbeen et al. J. Exp Med 2011, ].

European Journal of Immunology

Specific comments

Fig. 4 A and D. What are the mutation frequencies?

We are thankful for the opportunity to display our mutation frequency data, which we include as supplementary Figure 4.

What is the W:S ratio?

We adhered to the IUPAC-conforming nomenclature, but we recognize that this might not be entirely intuitive. We have now labeled the W:S ratio as (AT):(GC) ratio, where the numerator indicates the frequency of mutations at A/T pairs and the denominator that at G/C pairs, in both cases after correction for base composition.

Fig. 4 C. What is the significance (p-value) of the drop in A:T mutations in JH4 between those found in Ung and Smug/Ung cells? The data for Jk5 looks like there is no difference, so it would not be “broadly similar” to the JH data (p. 11, line 15). Also on p. 14, line 15, “small but convincing” (assuming the statistics hold up) does not apply to the Jk5 data.

As requested by all reviewers we now present statistical analyses for all the data within the manuscript figures. We now also include in Figure 4F a table summarizing statistical evaluation of the mutation distribution (counts per genotype) across the four bases using Fisher’s exact test.

We fear the use of the unqualified “broadly similar” expression in our previous version of the manuscript lead to misunderstanding, while it was meant to indicate a similar reduction of the AT ratio with removal of the uracil glycosylases (which is a novel observation) in the kappa locus. As we now show in detail in Supplementary Figure 4, we do indeed find a decrease in the mutation frequency at A:T base pairs when comparing *Smug1*$^{-/-}$*Ung*$^{-/-}$ mice to single *Ung*$^{-/-}$ mice. In both the JH4 and Jκ5 datasets, we find statistically significant differences ($p<0.05$) in the frequency of mutations at both A and T in uracil excision deficient cells ($S^-U^-$) compared to wildtype cells while no effect is observed at C or G bases.

These data are condensed in Fig4 C as ratios of mutation frequencies at A/T versus C/G pairs. Again the statistical evaluation supports our conclusion that differences in ratios in uracil excision competent versus the double KO cells are biologically relevant, lending strong support to our conclusion that uracil excision is contributing to the MSH2 dependent phase 2 of hypermutation. We do detect a further decrease in the A/T to G/C mutation ratio in the *Smug1*$^{-/-}$*Ung*$^{-/-}$ double deficient cells compared to the single *Ung*$^{-/-}$, which is readily evident only in the highly mutated JH4 sequences.

We have now modified the paragraph to improve the overall clarity of the text.

Fig. 4 D and E are not mentioned the text.

Figure 4 D and E were only summarily mentioned as part of Fig 4; we now specifically refer to them when talking about the Jk5 data.

European Journal of Immunology

Reviewer: 2

Comments to the Author

This paper demonstrates that Smug1, a uracil glycosylase, contributes to both Ig class switch recombination (CSR) and somatic hypermutations (SHM) in B cells lacking UNG.  It might make a small contribution even in UNG-proficient cells but it is much less important than UNG in excising dUs introduced by AID activity.  The study is complete and the discussion of the literature and their own data are excellent.  This is an important contribution about a complicated and somewhat controversial subject.

My criticisms are minor.

I think they should determine if CSR to IgG3 and IgG2b are decreased in the absence of Smug1.  I think IgG1 is the wrong isotype.  This is not terribly important in the context of the entire manuscript, which clearly shows that Smug1 has a small effect when UNG is missing, but it would have been interesting to see if Smug1 deficiency affected isotypes that are likely to be more dependent on the frequency of DNA breaks.

We agree with the reviewer and indeed performed in vitro class switch experiments to other isotypes, however we found no differences between control and *Smug1*$^{-/-}$ cells (assayed at day 5 of culture). We now mention this in the manuscript.

They should provide the frequency of SHM observed in the 4 different genotypes they study for the Jh4 and Jk5 introns.

This is an important point that we omitted in our initial submission in the interest of brevity.  As discussed in response to reviewer 1, we now present the frequencies in Supplementary Figure 4, and discuss the data in the main text of the manuscript.

They should also provide a reference for their statement that Sg1 is the most repetitive and hotspot-enriched switch region (p. 12).  My information disagrees with this statement, although since Sg1 consists of both repetitive and non-repetitive sequences, it is possible that there is a calculation for hoptspot density that excludes the latter regions and this gives rise to their statement.  This should be referenced.

We apologize for this statement that was again unqualified.  We based our initial statement on the lenght of the main repetitive region in the gamma 1 switch region (

2301 bp at Chr12:113334624-113336924 Consensus CTGCTCTGCCTGGGTCACCACACTTCCACCTGTCCTGGCTGCCCTGTAG versus gamma 3 (1637bp at Chr12:113363458-113365094 Consensus AGCTGCCCAGCCTGGTCCCCATACCCCCACCTACCCCAGCTCCCCCAGAGCTGCCCAGCCTGGTCCCCACACCCCACCTACCCCAGCTCCCCCAG).  The repeats in gamma 2b are discontinuous while the main repeat region in gamma2c is 2216 bp (Chr12:113293140-113295355 Consensus CTGTACTGCCTGGTCCCTACCCACAG) but appears to contain fewer potential WRCW hot spot consensus.  The repeats in the mouse alpha region are again discontinuous, with two main tandem repeat regions of 492 and 661 bp in length.  The consensus and lengths are based on the Ensembl mouse genome build GRCm38.  We have modified the text to qualify this statement, and refer the reader to the genome build.  A few studies have addressed the influence of the repeats and hot-spot density (for example the Yu and Alt labs) however to our knowledge, no definitive direct comparison has been made between the different mouse switch regions, although the available data suggests a role for both the length and the density of hot-spots in promoting efficient class switching.

European Journal of Immunology

Reviewer: 3

Comments to the Author

In both the title and abstract of this manuscript, the authors conclude that SMUG1 contributes to efficient Ig class switching. However, none of the data in the manuscript support this conclusion.

As we clearly state in the abstract, the emphasis of our manuscript is on the contribution of endogenous SMUG1 to antibody diversification. Critically, our experiments comparing SMUG1/UNG double-deficient and UNG-deficient animals show that endogenous SMUG1 is necessary for efficient function of the previously recognized MSH2-dependent pathway and contributes to class switching and hypermutation. Interestingly, serum titers of several immunoglobulin isotypes are not strongly affected by isolated deficiency of either glycosylase, but greatly diminished when both are removed, so SMUG1-catalysed switching is at least in this sense **efficient**, and in the case of IgG2b can rival that observed in the presence of UNG.

We have made a change in the title of the paper that we hope will reduce the likelihood of misinterpretation and that reflects our conclusion that endogenous SMUG1 acting on uracil at Ig locus (on the occasion that is able to do so) does "promote" efficient class switching.

The data do suggest that SMUG1 might provide a low “backup” level of CSR in situations where UNG is absent.

As we discuss extensively in our manuscript, the relative contributions of the different pathways of antibody diversification are highly dependent on the chosen readout and time of analysis. For instance, in the case of IgG2b, Smug1 seems able to fully compensate for UNG deficiency as regards serum levels at 26 weeks; compensation for other isotypes is only partial (but can be substantial). Thus, the backup is not necessarily “low” even in absolute terms.

However, there is no statistical evaluation of the data supporting this “backup” role, so even this limited function has not been demonstrated.

As discussed in response to the other reviewers, we have now presented the statistical analysis of our data.

If the manuscript is revised to include the requisite statistical evaluation of the SMUG1 CSR function, it is still not clear that the demonstration of this “backup” CSR activity would be sufficiently important to a broad immunological audience to warrant publication in EJI.

By demonstrating that when endogenous Smug1 excises uracils it promotes efficient class switching, our data show that uracil removal is the critical step initiating class switching, rather than a privileged role ascribable to UNG. As highlighted by reviewers 1 and 2, our manuscript clarifies the role of base excision in both CSR and SHM and raises the question of the mechanistic source of breaks that promote mismatch repair-induced mutations at A:T pairs.

We feel this should be a welcome contribution to the field, and have specifically identified EJI as a journal that may convey our mechanistic insights to a broader immunological audience.

It is also surprising that the authors did not use a digestion-circularization PCR approach to directly measure the production of CSR DNA joins in cells stimulated to switch in culture; instead relying on FACS measurement of the production of surface isotype-switched Ig proteins (which assumes that transcription, translation and surface deposition of protein provide a directly linear relationship with DNA recombination events).

European Journal of Immunology

We thank Reviewer 3 for this interesting suggestion. Digestion-circularisation PCR for the measurement of switch junctions was originally described in 1992 by Chu and colleagues, who describe it as “sensitive enough to quantitate switched cells constituting only 1-2% of the population”. In 1993, the same authors demonstrated that the methods correlates well with surface IgG1 expression, except for a few cases where “measurement inaccuracies” may account for the difference, since the technique is “near [its] limit of sensitivity” at rearrangement frequencies of 2.5 – 3.8 %. We note that switching in the absence of UNG is below this level.

We have attempted a modified Q-PCR assay that relies on limiting dilution to try to extend the sensitivity of this assay as applied to very small populations of switched cells (an example is shown below). While the results are consistent with our flow cytometry observations, the method would require a high number of replicates to reach similar quantitation accuracy as modern flow cytometry where rapid acquisition of large number of events with little optical noise routinely permits accurate quantitation of rare populations, evidenced in our case by the highly reproducible results in *Smug1*$^{-/-}$ *Ung*$^{-/-}$ mice (Fig. 3). We feel that switched immunoglobulin protein is the relevant outcome of successful class switch recombination, and measuring it strengthens rather than weakens our conclusion.

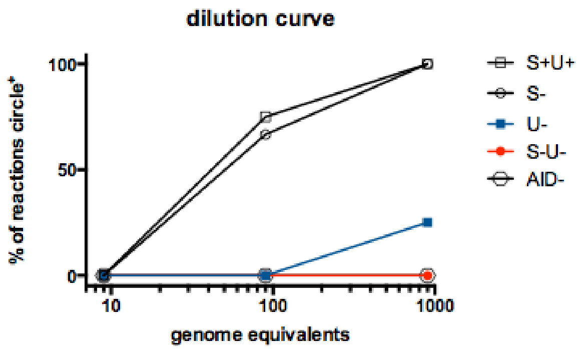


Results from 3 replicates per genotype expressed as the % of positive reactions. Briefly, DNA was prepared from LPS/IL4 induced cultures after 3 days and processed as originally described by Chu et al. 1993, J Exp Med 178:1381-1390, but with primers and probes adapted for Q-PCR. Each sample was assayed at three 10-fold dilutions (plotted as genome equivalents).

The following figures require statistical analyses to show whether differences noted in the text between US and U mice are significant: Fig 2, Fig. 3, Fig. 4, Sup. Fig. 3.

As mentioned before, we have included the statistical evaluation and the tests are described within the figure legends.

The methodology for obtaining the mutant mice born from AID-/- mothers should be briefly described.

We apologize for the omission of details of the breeding strategy which is now included in the materials and methods section.

# Peer review correspondence

**Second Editorial Decision – 8 April 2014**

Dear Dr. Rada,

It is a pleasure to provisionally accept your manuscript entitled "Uracil excision by endogenous SMUG1 glycosylase promotes efficient Ig class switching and impacts on A:T substitutions during somatic mutation" for publication in the European Journal of Immunology. For final acceptance, please follow the instructions below and return the requested items as soon as possible as we cannot process your manuscript further until all items listed below are dealt with.

Please note that EJI articles are now published online a few days after final acceptance (see Accepted Articles: http://onlinelibrary.wiley.com/journal/10.1002/(ISSN)1521-4141/accepted). The files used for the Accepted Articles are the final files and information supplied by you in Manuscript Central. You should therefore check that all the information (including author names) is correct as changes will NOT be permitted until the proofs stage.

We look forward to hearing from you and thank you for submitting your manuscript to the European Journal of Immunology.

Yours sincerely,
Laura Soto Vazquez

on behalf of
Prof. Hans-Martin Jack

Editorial Office
European Journal of Immunology
e-mail: ejied@wiley.com
www.eji-journal.eu
